# Identification of Prognostic Biomarkers for Suppressing Tumorigenesis and Metastasis of Hepatocellular Carcinoma through Transcriptome Analysis

**DOI:** 10.3390/diagnostics13050965

**Published:** 2023-03-03

**Authors:** Divya Mishra, Ashish Mishra, Sachchida Nand Rai, Emanuel Vamanu, Mohan P. Singh

**Affiliations:** 1 Centre of Bioinformatics, Affiliated University of Allahabad, Prayagraj 211002, India; 2 Centre of Biotechnology, Affiliated University of Allahabad, Prayagraj 211002, India; 3 Faculty of Biotechnology, University of Agricultural Sciences and Veterinary Medicine, 011464 Bucharest, Romania

**Keywords:** hepatocellular carcinomas (HCC), glioblastoma multiforme (GBM), metastasis, RNA-seq analysis, hub gene, cox regression analysis, GEPIA

## Abstract

Cancer is one of the deadliest diseases developed through tumorigenesis and could be fatal if it reaches the metastatic phase. The novelty of the present investigation is to explore the prognostic biomarkers in hepatocellular carcinoma (HCC) that could develop glioblastoma multiforme (GBM) due to metastasis. The analysis was conducted using RNA-seq datasets for both HCC (PRJNA494560 and PRJNA347513) and GBM (PRJNA494560 and PRJNA414787) from Gene Expression Omnibus (GEO). This study identified 13 hub genes found to be overexpressed in both GBM and HCC. A promoter methylation study showed these genes to be hypomethylated. Validation through genetic alteration and missense mutations resulted in chromosomal instability, leading to improper chromosome segregation, causing aneuploidy. A 13-gene predictive model was obtained and validated using a KM plot. These hub genes could be prognostic biomarkers and potential therapeutic targets, inhibition of which could suppress tumorigenesis and metastasis.

## 1. Introduction

Cancer is a complex disease caused due to uncontrolled division and growth of cells, categorized according to the progression in organs such as breast cancer, blood cancer, colon cancer, liver cancer, etc. Liver cancer, also known as hepatocellular carcinoma (HCC), has nowadays become a common cancer type, with approximately 830,000 deaths in 2020 alone [1]. Tumorigenesis or the transformation of normal cells into cancerous cells often results in uncontrolled cell proliferation, metastasis, and apoptosis evasion [2]. Metastasis of cancer cells occurs through blood vessels and lymph nodes and accounts for the development of other types of cancers [3]. Its occurrence is common in hepatocellular carcinoma (HCC) patients undergoing surgery [4]. Most of the patients are diagnosed during the late phase of the disease. Significant advancements in early disease diagnosis through standard interventions such as radiation, surgery, personalized strategies, and chemotherapy have been developed in the past few decades. The aggregative five-year survival rate of HCC and GBM remains destructive due to their molecular heterogeneity and invasive behavior. It has been found in some studies that brain metastases from HCC are less frequent (0.2–2.2%) and resulted in poorer survival of patients [5]. Even though this rate is less, but still it is certain that 10–45% of different cancer types from liver, lung, and other body parts metastasize to the brain [6]. This process of metastasis can be dealt with using biomarkers. Biomarkers are biological molecules found in body fluids, blood samples, or tissues and these signify a particular condition or disease. In different cancer types, metastatic biomarkers help in detecting the early stages of tumor spread and recurrence probability and in predicting the favorable sites of metastasis [7]. Once metastatic cancer is detected, further we need to identify the DNA or RNA-based biomarkers that could allow for personalized therapy resulting in significantly positive survival outcomes in patients [8]. Some biomarkers such as CDK4, PTEN, and ERBB2 were found as potential indicators for the diagnosis and prediction of metastatic breast cancer [9]. Likewise, overexpression of EGFR in metastatic non-small cell lung cancer (NSCLC) makes it a prognostic biomarker [10]. In the past few years, the fast growth of in silico approaches such as next-generation sequencing has enabled insight into carcinogenesis and progression of distinct cancer [11]. High-throughput platforms have been extensively used in prognosis prediction, histological identification, early diagnosis, disease resistance analysis, and molecular classification. Long noncoding RNAs (lncRNAs), microRNAs (miRNAs), differentially expressed genes (DEGs), and differentially methylated CpG sites can potentially serve as valuable HCC biomarkers [12]. Few oncogenic lncRNAs such as LASP1-AS, MALAT1, HOTAIR, and NORAD acted as potential biomarkers in the case of HCC. Similarly, some tumor-suppressor genes viz. DGCR5, MIR22HG, and HOTAIRM1 are also found as potential biomarkers for HCC [13]. An example of miRNAs includes miR-125a-5p which was upregulated in patients having HCV-associated hepatocellular carcinoma [14]. Similarly, the overexpression of some differentially expressed genes such as CDC20, BUB1B, AURKA, CCNA2, and BUB1 was found responsible for the poor progression and high mortality of patients suffering from HCC [15]. The transcriptome analysis has disclosed the cancer molecular mechanisms. Meanwhile, few reports have been introduced to identify the candidate biomarkers related to HBV-HCC with combined datasets [12]. In this study, the transcriptome analysis was carried out using RNA-seq datasets to identify the differentially expressed genes (DEGs) that play a vital role as potential prognostic biomarkers in the case of metastatic HCC and GBM. One of the most important biological processes obtained from the DAVID database using DEGs, chromosome segregation, has a prominent role in tumorigenesis as any chromosomal instability causes genetic instability due to dysregulated chromosome segregation [16]. Similarly, the cell cycle, which is an important KEGG pathway, was obtained. Any aberrant change in this cycle may also result in tumorigenesis. Hence, its regulators could be treated as potential anticancer therapeutic targets [17].

Another predominant feature related to tumor development and progression is an alteration in DNA methylation. DNA hypomethylation is more prominent with tumorigenesis or malignancy than hypermethylation [11,18,19]. It has been found that genomic instability occurs due to DNA hypomethylation in the case of HCC [20,21,22]. This instability causes the activation of oncogenes such as antigen family A1 (*MAGEA1*) [23]. Genetic alterations in the form of mutations and DNA copy number alterations (CNAs) were also identified as critical features of HCC tumorigenesis and metastasis. A study found that missense mutation in the NUF2 gene was linked to cancer development and hence, its inhibition resulted in the suppression of tumor growth leading to cancer cells apoptosis [24]. Copy number alterations are present in 90% of solid tumors and play a prominent role in activating oncogenes and inactivating tumor suppressor genes by altering the dosage and structure of genes [25]. As CNAs outline pivotal genetic events that drive tumorigenesis, such genetic alterations have the potential as predictive factors [26]. Post-translational modifications (PTM) viz. phosphorylation, acetylation, Ubiquitination, methylation, sumoylation, etc., also play a vital role in the tumorigenesis of different cancer types, particularly in breast cancer [27]. Mutation in Aurora Kinase A (AURKA) in HCC through direct phosphorylation of Pkinase promoted tumorigenesis and subsequently metastasis [28]. Likewise, Ubiquitination, another PTM plays a vital role in administering the control of substrate degradation, which is required for the proper functioning of the cell cycle, and any aberrancy in this process will hamper normal cell functioning leading to cancer development and possibly metastasis [29]. In this study, aberrant Ubiquitination in Pkinase led to mutations in the AURKA gene and this abnormal overexpression resulted in tumorigenesis and later-stage metastasis of HCC. This study, therefore, involved the identification of differentially expressed genes that were overexpressed in both GBM and HCC. The 13 hub genes obtained were further validated through promoter methylation, mutation, and genetic alterations analysis proving their potential to be prognostic biomarkers. The survival analysis of all these hub genes showed poorer survival rates among metastatic HCC and GBM patients.

## 2. Materials and Methods

**Figure 1:**
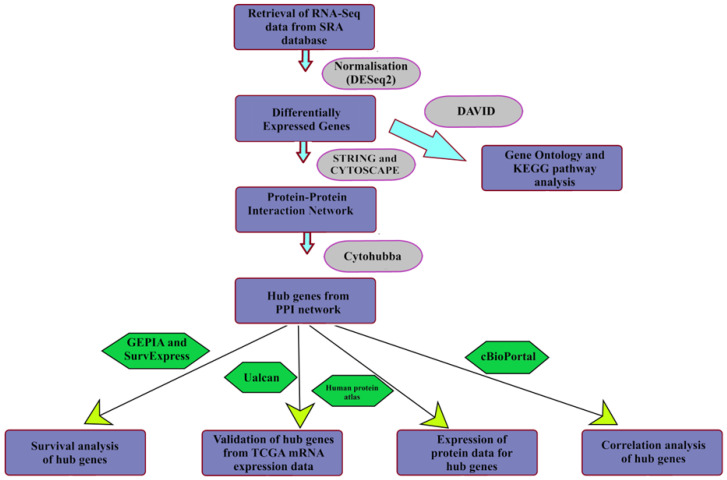
Flowchart showing the methodology followed in the present study.

The datasets for both GBM and HCC were taken from Gene Expression Omnibus (GEO). For GBM (normal samples-PRJNA494560 transcriptomic data with paired-end sequencing performed on Illumina HiSeq 3000 (Homo Sapiens) platform and tumor samples-PRJNA347513, transcriptomic data with paired-end sequencing performed on Illumina HiSeq 2000 platform) and for HCC (normal samples-PRJNA494560 transcriptomic data with paired-end sequencing performed on Illumina HiSeq 3000 (Homo Sapiens) platform and tumor samples-PRJNA414787 transcriptomic data with paired-end sequencing performed on Illumina HiSeq 2000 (Homo Sapiens) platform) were taken. The method that was followed for carrying out this study is shown below in flowchart (see Figure 1).

### 2.1. Identification of Common Prognostic Biomarkers in HCC and GBM

#### Data Pre-Processing

The data pre-processing of the raw reads was performed on Galaxy, an open-source platform for analyzing genomic data [30]. Galaxy implements FastQC (Version 0.11.8), a quality control tool for high-throughput sequenced data, for conducting the quality assessment of raw reads and removing the adapter sequences, uncalled bases and low-quality reads for improving the sequence quality through filtering and trimming. For this purpose, Cutadapt tool (v 3.2) is implemented. After obtaining the high-quality data from pre-processing, the next step that follows is alignment of reads against human reference genome (GRch38/hg38). This is accomplished using STAR (Version 2.7.7a) which is an ultrafast universal RNA-seq alignment tool [31,32]. The mapped reads are subsequently quantified in a process called quantification, through featureCounts (subread Version 2.0.1) package [33]. This step provides read counts per annotated gene. The normalized read count is further taken and, eventually, the statistical analysis is performed to obtain the differentially expressed genes (DEGs) between control and treated groups. It provides the quantitative changes in expression levels of genes. DESeq2 (Version 1.22.1) is a tool that performs this normalization process and is based on negative binomial distribution [34]. DEGs having FDR (*p*-value (adj)) <0.05 and |Log2FC| > 2 are considered statistically significant.

### 2.2. Protein-Protein Interaction Network Analysis and Identification of Hub genes

The protein-protein interaction network is critical for understanding the cellular processes in diseased and normal states. This network provides the mathematical representations of the physical contacts between different proteins. This network was obtained by taking DEGs as input in the STRING database [35]. The vertices constitute DEGs (proteins) and edges constitute the protein interactions. The network was subsequently visualized through Cytoscape software [36]. The confidence score was taken <0.4. The PPI enrichment value less than 1 × 10^−16^ indicated that the network has significant interactions. The modular analysis was obtained by implementing MCODE (Molecular Complex detection) plug-in of Cytoscape. The parameters included degree cut-off = 0.2, node score cut-off = 0.2, k-core = 2, and maximum depth = 100. The hub genes are then identified from the obtained module using cytohubba plug-in. Hub genes are hugely interconnected genes and play a critical role in PPI network. For this purpose, 5 different topologies i.e., Maximal Clique Centrality (MCC), Degree, Edge Percolated Component (EPC), Maximum Neighborhood Component (MNC), and Radiality were employed. The topmost 15 were considered as hub genes in all the 5 algorithms and the common 13 hub genes were then taken from these 5 topologies through venn diagram obtained from jvenn [37].

### 2.3. GO Component and Pathway Enrichment Analysis

Both GO and KEGG pathway enrichment analysis was obtained by providing common DEGs between GBM and HCC as input to DAVID database which is an online tool for functional enrichment analysis [38]. For both GO term and KEGG pathway, the EASE value (modified Fisher Exact *p*-value), employed for measuring the gene enrichment in annotation terms, was set to 0.1 and the count threshold to 2 (default value). The lesser this *p*-value is, the more enriched the GO terms or KEGG pathways are. The cut-off value for any term or pathway to be significant was set at *p* < 0.05. REVIGO [39] was used subsequently for constructing the treemap for biological processes by entering GO ids of all the terms along with their respective *p*-values.

### 2.4. Epigenetic Regulation of Gene Expression of Hub Genes by Promoter Methylation

DNA methylation is an epigenetic factor that plays a crucial role in gene regulation. It is a feature of different types of human diseases and is predominant in case of different cancer types. The epigenetic alterations have an effect on the genes participating in the tumorigenesis and metastasis of cancer [40]. In this study, UALCAN [41], which is an online web resource for the analysis of cancer OMICS data, was employed for obtaining the promoter methylation of hub genes through TCGA datasets for both GBM and HCC. The beta values indicated DNA methylation levels ranging from 0 (i.e., unmethylated) to 1 (i.e., fully methylated). For hypermethylation, the specified range of beta value was 0.5–0.7, and, for hypomethylation, this range was 0.05–0.3.

### 2.5. Genetic Alterations of Hub Genes

The genetic alterations that mainly include mutations and DNA copy number alterations correspond to changes in the DNA sequences due to various factors. The accumulation of such genetic alterations may lead to cancer development, metastasis, growth, and resistance to therapy. This validation of genetic alterations in the hub genes was accomplished through cBioPortal [42], which is an open-source, open-access resource for interactively exploring multidimensional cancer genomics data sets. For this purpose, 592 TCGA samples were considered for GBM and 391 samples for HCC. Copy number data sets were generated via GISTIC (Genomic Identification of Significant Targets in Cancer) algorithms that identify those regions that are significantly altered across the sets of patients. OncoPrints are used for visualization of the genomic alterations (mutations and copy number alterations) and mRNA expression changes across a set of TCGA cases for the hub genes. In case of mutations, a splice site mutation occurs in an intronic region while splice region mutations take place near the exon/intron junction. The copy number analysis derived from GISTIC algorithms indicates the level of copy number per gene. In this case, −2 indicate deep deletion or deep loss and correspond to homozygous deletion. Notably, −1 corresponds to shallow deletion and indicates a heterozygous deletion. Notably, 0 is assigned to normal or diploid. Notably, 1 corresponds to gain which indicates low-level gain and 2 correspond to amplification which indicates a high-level amplification.

### 2.6. Differential Expression Pattern Validation and Survival Analysis of Hub Genes

The gene expression profiles of normal and cancerous TCGA samples related to all the 13 hub genes in both GBM and HCC were obtained through GEPIA (Gene Expression Profiling Interactive Analysis), an online web server [43]. Thereafter, the survival analysis of these hub genes was obtained via the web-based tool, SurvExpress [44]. The TCGA dataset in this case contained 148 patient samples of GBM and 361 patient samples of HCC. The univariate cox regression analysis was employed to obtain the risk score by grouping the patients into high- and low-risk groups. Further, the Kaplan-Meier plot was obtained for visualizing the survival analysis of all the 13 hub genes (potential biomarkers) in both GBM and HCC.

## 3. Results

### 3.1. Differentially Expressed Genes

There are a total of 3265 differentially expressed genes (1570 upregulated and 1695 downregulated) obtained from GBM datasets and 2321 differentially expressed genes (1444 upregulated and 877 downregulated) from HCC (see Supplementary Figure S1). The normal and cancerous tissues of the brain (GBM) and liver (HCC) cancer are taken from the Human Protein Atlas (HPA) (see Supplementary Figure S2). Out of these differentially expressed genes obtained for both GBM and HCC, there are 757 differentially expressed genes (452 upregulated and 305 downregulated) that are shared between both GBM and HCC. These 757 differentially expressed genes are considered for further analysis of network and pathway enrichment. These are common DEGs that are taken forward from the same NGS-analyzed data.

### 3.2. Protein-Protein Interaction Network Analysis

**Figure 2:**
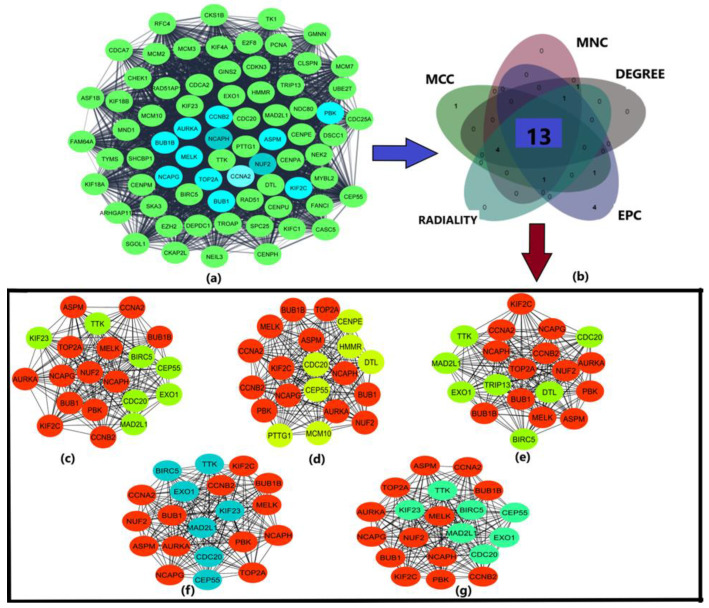
(**a**) Module of the PPI network obtained from MCODE plug-in of Cytoscape, (**b**) Venn diagram obtained from jvenn to find the common hub genes, (**c**) 13 hub genes using degree method of cytohubba shown in red, and green color reparents interacting proteins (**d**) 13 hub genes using EPC method of cytohubba shown in red, yellow color represents the interacting proteins with the hub genes (**e**) 13 hub genes using MCC algorithm of cytohubba shown in red, light green color indicates the interacting proteins (**f**) 13 hub genes using MNC algorithm of cytohubba shown in red, teal green color represents the interacting proteins (**g**) 13 hub genes using radiality algorithm of cytohubba shown in red, green color represents interacting proteins.

The PPI network for the differentially expressed genes contained 757 nodes and 6628 edges (see Supplementary Figure S3). The PPI enrichment *p*-value was less than 1 × 10^−16^. Since this value is small, it indicates that the nodes are not random, and the observed number of edges is significant. The modules obtained from the MCODE plug-in and subsequently cytohubba plug-in provided 13 common hub genes in both GBM and HCC, viz Assembly Factor for Spindle Microtubules (ASPM), Aurora Kinase A (AURKA), BUB1 Mitotic Checkpoint Serine/Threonine Kinase (BUB1), BUB1 Mitotic Checkpoint Serine/Threonine Kinase B (BUB1B), Cyclin A2 (CCNA2), Cyclin B2 (CCNB2), Kinase Family Member 2C (KIF2C), Maternal Embryonic Leucine Zipper Kinase (MELK), Non-SMC Condensin I Complex Subunit G (NCAPG), Non-SMC Condensin I Complex Subunit H (NCAPH), NUF2 Component of NDC80 Kinetochore Complex (NUF2), PDZ Binding Kinase (PBK), and DNA Topoisomerase II Alpha (TOP2A) (see Figure 2). All these genes had a function associated with chromosome and spindle behavior of mitotic cell division and showed an up-regulated expression level in both GBM and HCC.

The pairwise correlation analysis of DEGs using Pearson correlation statistics showed a higher degree of positive correlations.

### 3.3. GO Component and Pathway Enrichment Analysis

**Figure 3:**
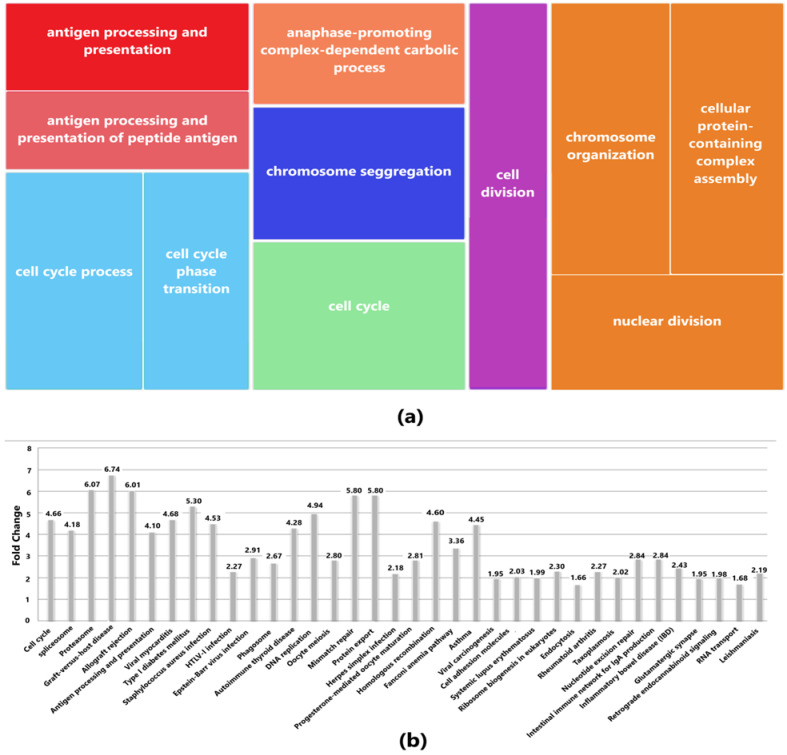
(**a**) Biological Processes (BP) based on *p*-values drawn from Revigo in which the hub genes are enriched. (**b**) KEGG pathways corresponding to the enrichment of hub genes based on *p*-values and fold change.

The results obtained for biological processes from the DAVID database showed that the hub genes are enriched in chromosome segregation, cell division, cell cycle process, nuclear division, and antigen processing and presentation. Likewise, the KEGG pathway analysis showed the involvement of 13 hub genes in the cell cycle, DNA replication, oocyte meiosis, progesterone-mediated oocyte maturation, viral carcinogenesis, and Epstein-Barr virus infection signaling pathways (see Figure 3).

### 3.4. Epigenetic Regulation of Gene Expression of Hub Genes by Promoter Methylation

Validation of promoter methylation using the UALCAN database revealed that the promoter methylation level of ASPM, AURKA, BUB1, KIF2C, NCAPG, NCAPH, and NUF2 was lower than normal samples in GBM that indicates higher expression of these hub genes as against that of BUB1B, CCNA2, CCNB2, MELK, PBK and TOP2A having higher promoter methylation level than normal samples (see Supplementary Figure S4a).

In the case of HCC, the expression level of BUB1, CCNA2, CCNB2, KIF2C, MELK, NCAPG, NCAPH, NUF2, PBK, and TOP2A was higher due to their lower promoter methylation level against normal samples while ASPM, AURKA and BUB1B were lowly expressed (see Supplementary Figure S4b).

### 3.5. Differential Expression Pattern and Survival Analysis validation of Prognostic Biomarkers

The differential expression between normal and tumor cells obtained from the GEPIA database showed that the expression of hub genes was significantly higher in the case of GBM as compared to HCC. Moreover, among the 13 hub genes, the expression level of the TOP2A gene was significantly higher in both GBM and HCC (see Supplementary Figure S5).

**Figure 4:**
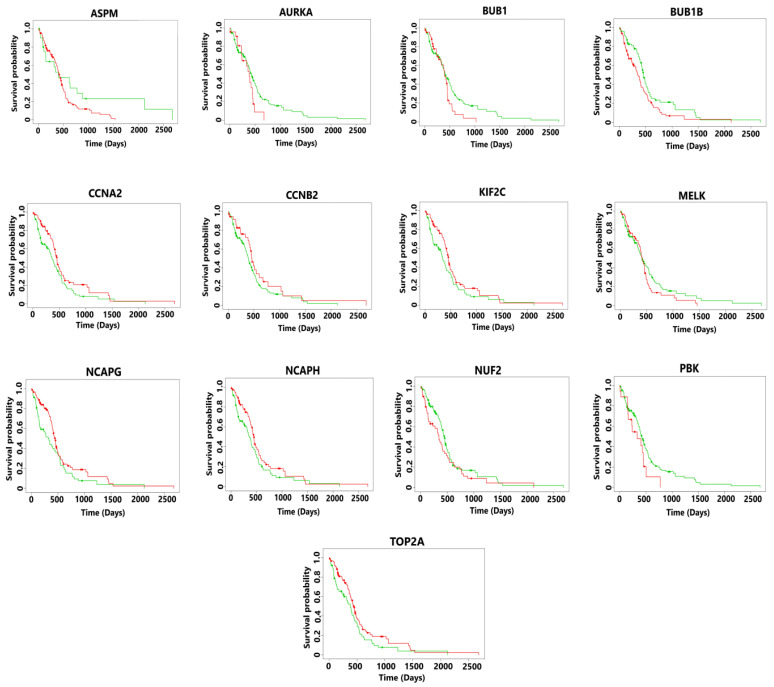
Kaplan-Meier plots showing the survival analysis of hub genes in GBM. The patients were divided into high- and low-risk groups. In this plot the green line in a survival curve typically represents the survival probability for a reference or control group, while the red line represents the survival probability for an experimental or treatment group. The overexpression of all the hub genes resulted in poor survival outcomes which are less than 2 years for the patients suffering from GBM.

The aberrant expression of ASPM, AURKA, BUB1, BUB1B, MELK, NUF2, and PBK resulted in a poorer survival rate of GBM patients in the high-risk group with a survival rate of fewer than two years. The median survival rate was less than 2 years for all the 13 hub genes (see Figure 4). For each patient, the risk score was calculated and ranking was carried out accordingly in the TCGA dataset. Patients were then divided into a high-risk group and a low-risk group.

Risk score (RS) = 0.03 * expression _ASPM_ + 0.041 * expression _AURKA_ + 0.044 * expression _BUB1_ − 0.03 * expression _BUB1B_ + 0.011 * expression _CCNA2_ + 0.007 * expression _CCNB2_ + 0.046 * expression _KIF2C_ + 0.009 * expression _MELK_ + 0.01 * expression _NCAPG_ + 0.013 * expression _NCAPH_ − 0.032 * expression _NUF2_ + 0.108 * expression _PBK_ + 0.035 * expression _TOP2A_

_In this formula, the asterisk symbol is being used to represent multiplication_

The hazard ratio > 1 for these hub genes also showed a higher level of survival risk (see Table 1).

**Figure 5:**
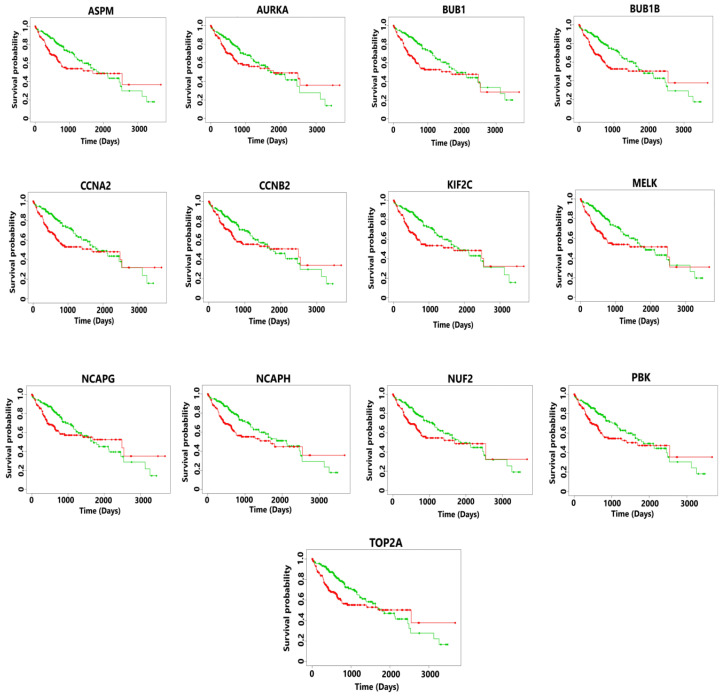
Kaplan-Meier plots showing the survival analysis corresponding to hub genes in HCC. The patients were divided into high- and low-risk groups. Here the green line in a survival curve typically represents the survival probability for a control group, while the red line represents the survival probability for an experimental or treatment group. The overexpression of all the hub genes resulted in poor survival outcomes which are less than 3 years for the patients suffering from metastatic HCC.

The survival analysis of patients in the high-risk group showed a poorer median survival rate which was less than 3 years (see Figure 5). The risk score was calculated as shown below.

Risk score (RS) = 0.078 * expression _ASPM_ + 0.02 * expression _AURKA_ + 0.25 * expression _BUB1_ + 0.268 * expression _BUB1B_ + 0.173 * expression _CCNA2_ + 0.139 * expression _CCNB2_ + 0.278 * expression _KIF2C_ + 0.278 * expression _MELK_ + 0.153 * expression _NCAPG_ + 0.235 * expression _NCAPH_ + 0.268 * expression _NUF2_ + 0.233 * expression _PBK_ + 0.062 * expression _TOP2A_

The hazard ratio >1 for all the 13 hub genes also indicated a poorer survival rate of HCC patients (see Table 2).

### 3.6. Genetic Alterations in Hub Genes

Tumorigenesis mainly occurs due to irremediable mutations in cell structures. These mutations could be identified through genetic alteration analysis. The alterations may be in the form of a missense mutation, splice mutation, deep deletion, truncating mutation, and amplification. In the case of GBM, the percentage alteration of all 13 hub genes varied from 0.3% to 2.1% (see Supplementary Figure S6). The corresponding copy number variations are shown in Supplementary Figure S7. The details of genetic alterations and copy number variations can be found in the table (see Supplementary Table S1). Most of the mutations in hub genes were found at phosphorylation, acetylation, and uniquitination PTM sites with the characteristics of missense mutation and diploid copy type alteration.

In the case of HCC, the alteration percentage had variations between 0.3–10% for the 13 prognostic biomarkers (see Supplementary Figure S8). The copy number variations are shown in Supplementary Figure S9. The description of genetic alterations and copy number variations are summarized in Supplementary Table S2. The results show that mutations mainly occurred at phosphorylation and ubiquitination PTM sites with diploid copy number variations and missense mutations and these features were found to be enriched in the tumorigenesis and metastasis of cancers with markedly stronger accumulation and evolutionary conservation in protein domains [45].

## 4. Discussion

Cancer development due to uncontrolled cell division is the leading cause of death worldwide. The most dangerous event that leads to cancer development is mitosis having the irreversible segregation of sister chromatids to daughter cells [46]. Abnormal chromosome segregation during mitosis results in tumorigenesis. This happens mainly due to failure in the mechanism of the spindle assembly checkpoint, as the checkpoint ensures proper chromosome segregation during mitosis [47]. This chromosomal instability that results in abnormal chromosome numbers produces uncontrolled cell division, leading to tumorigenesis and subsequently to the metastasis of cancer types [48]. Hepatocellular carcinoma (HCC) is one of the leading cancer types that metastasizes to the lungs, adrenal glands, lymph nodes, and brain [49]. The evidence of brain metastasis from HCC is rare but is nowadays becoming more frequent compared to the conditions in the past [50]. This study mainly focused on the metastasis of HCC to the brain that could potentially lead to the development of glioblastoma multiforme (GBM), the IV grade brain cancer. This progression and metastasis of this cancer resulted from the aberrant function of some genes and alteration in the patterns of gene expression. This dysregulation in the gene expression is mainly due to genetic alterations such as mutation, amplification, and copy number alterations [51]. Thirteen hub genes were obtained from the protein-protein interaction network analysis using differentially expressed genes. Similarly, the pathway enrichment analysis carried out using the DAVID database showed the involvement of these hub genes in processes such as cell cycle, cell cycle process, and oocyte meiosis. These signaling pathways actively participate in cancer development, leading to tumorigenesis and metastasis.

Further validation of these genes was carried out using the UALCAN database which found them to be hypomethylated in both HCC and GBM. This resulted in their aberrant expression through the increased probability of undergoing mutations leading to tumorigenesis [52]. All the 13 hub genes, i.e., ASPM, AURKA, BUB1, BUB1B, CCNA2, CCNB2, KIF2C, MELK, NCAPG, NCAPH, NUF2, PBK, and TOP2A are oncogenes and were found to be upregulated in all the samples of HCC and GBM. ASPM gene had abnormality due to its overexpression in HCC and played a vital role in cell proliferation and metastasis (Lin et al., 2008). It promotes the progression of HCC through the activation of Wnt/β-catenin signaling [53]. This gene had missense mutations at eight different locations in two percent of the patients on phosphorylation and ubiquitination post-transcriptional modification (PTM) sites (Supplementary Table S2). These mutations happened due to diploid copy number alteration. Likewise, in the case of GBM, it had missense mutations on phosphorylation, acetylation, ubiquitination, and methylation PTM sites at 17 different locations (Supplementary Table S1). Aurora Kinase A (AURKA) also has tumorigenesis properties in different cancer types [54]. This gene was involved in cancer metastases in the case of HCC [55]. The missense mutation having diploid type alteration in 0.57% of the patients on phosphorylation PTM sites at 2 locations resulted in the abnormality in this gene. Likewise, in the case of GBM, this gene has missense mutations in 0.57% of the patients, with diploid copy number alterations on phosphorylation and acetylation PTM sites at 3 different locations. In one of the studies, it was found that AURKA inhibition suppressed the cell proliferation of GBM [56]. BUB1 overexpression promoted tumorigenesis and aneuploidy [57]. This resulted in poorer survival of patients suffering from HCC (Yang et al., 2019). It had missense mutations in 0.57% of the patients at 2 different locations with diploid copy number alterations. In GBM also, upregulated BUB1 was also responsible for cell proliferation resulting in tumorigenesis [58]. It has missense and splice mutations at five different locations. It has phosphorylation and ubiquitination PTM sites with diploid and shallow deletion type of copy number alterations. The next hub gene BUB1B was involved in the progression of hepatocellular carcinoma (HCC) by activating mTORC1 signaling pathway [59]. It has a missense mutation at a single location in 0.29% of the patients. It has diploid copy number alterations. In the case of GBM, BUB1B was found to promote tumor proliferation [60]. It has splice mutation in 0.29% of the patients at a single location and has diploid copy number alterations associated with it. CCNA2 is found to promote uncontrolled cell growth, resulting in tumorigenesis in the case of different cancer types [61,62,63]. The upregulated CCNA2 was involved in cell cycle progression that resulted in tumorigenesis and metastasis in the case of HCC [64]. It had a missense mutation in 0.29% of the patients with diploid copy number alterations. In the case of GBM, overexpressed CCNA2 resulted in a poor prognosis for patients [65]. It had missense and nonsense mutations in 0.29% of the patients on acetylation, phosphorylation, and ubiquitination PTM sites on A25V and E269. CCNB2 was also found to promote cell cycle progression resulting in tumorigenesis in cases of triple-negative breast cancer [66]. It was also identified in the cell cycle progression leading to poor prognosis of HCC [67]. In this study, amplification was found as genetic alterations in 0.57% of the patients. In GBM, CCNB2 acted as a potential biomarker and played a vital role in Cellular Senescence and cell cycle [68]. These have missense mutation at location P80S having phosphorylation PTM site and amplification in 0.57% of the patients and shallow deletion copy number alterations. The absence of mutation in HCC and the presence of one mutation in GBM showed that this mutation might have taken place due to metastasis of HCC in the brain leading to GBM. KIF2C resulted in tumorigenesis due to abnormal cell cycle progression and metastasis in cervical cancer [69]. In the case of HCC, it participated in the progression of HCC and could be a potential therapeutic target [69]. On the other hand, in the case of GBM, it had missense mutations at three different locations on phosphorylation, ubiquitination, acetylation, and methylation PTM sites with diploid and gain copy number alterations. According to a study, this MELK gene was found to possess therapeutic drug-like properties due to its role in cell proliferation and triggering of cell cycle arrest in different cancer types [70]. Its overexpression in the case of HCC strongly correlated with abnormal cell growth leading to early recurrence and poor prognosis of patients [71]. In the following study, it had missense mutations in 1.43% of the patients at 4 different locations on phosphorylation PTM sites having diploid copy number alterations. In GBM, MELK developed tumorigenesis and its inhibition could effectively suppress the abnormal growth of GBM [72]. Here, it was found to have missense mutations in 1.43% of the patients. It has phosphorylation PTM site and diploid copy number alterations. NCAPG gene was found to be responsible for the survival of tumor cells leading to tumorigenesis and metastasis in HCC [73]. It had missense and nonsense mutations in 0.86% of the patients at 3 different locations. It had diploid copy number alterations on phosphorylation and ubiquitination PTM sites. Similarly, NCAPG was responsible for promoting tumor progression in the case of GBM also [74]. It had missense, splice, and nonsense type mutations in 0.86% of the patients at 5 different locations on phosphorylation, ubiquitination PTM sites and diploid, gain, and shallow deletion copy number alterations. NCAPH was found to be overexpressed in different cancer types promoting tumorigenesis and possibly metastasis [75,76,77,78]. The upregulation of NCAPH resulted in the enhancement of cell proliferation, invasion, and migration in the case of HCC [79]. In this study, it had missense mutations in 0.29% of the patients having diploid copy number alterations. In the case of GBM, it had missense mutations in 0.29% of the patients with diploid copy number alterations. This gene played a regulatory role in cell proliferation and apoptosis in the case of HCC [80]. It had missense mutation at 3 locations in 0.86% of the patients having the gain type of copy number alterations. In GBM, NUF2 promoted tumorigenesis and its downregulation inhibited the growth of tumor cells and induced apoptosis [81]. It had a missense mutation in 0.79% of the patients at three different locations on phosphorylation and ubiquitination PTM sites having diploid copy number alterations. The overexpression of PBK in the case of HCC promoted metastasis through activating the ETV4-uPAR signaling pathway [82]. It had a missense mutation at E303V in 0.29% of the patients having diploid copy number alterations in HCC. Similarly, its overexpression resulted in a poorer survival rate in the case of GBM [83]. It had missense mutations in about 0.26% of the patients on phosphorylation PTM sites having diploid copy number alterations. TOP2A was associated with growth in HCC tumor cells resulting in metastasis [84,85]. It had nonsense and missense mutations in 1.14% of the patients at 4 different locations on phosphorylation PTM sites having diploid and gain copy number alterations. In the case of GBM also, TOP2A had missense mutations at 5 different locations in 0.79% of the patients on phosphorylation, sumoylation, acetylation, ubiquitination, and methylation PTM sites having diploid copy number alterations. These 13 hub genes that were discussed above were associated with the worst survival of the patients as studied through Kaplan-Meier survival plots in the case of both GBM and HCC. This survival rate was less than two years due to overexpression of these genes, and hence these could be potential prognostic biomarkers that could help in the suppression of metastasis of HCC.

## 5. Conclusions

The present study identified 13 gene signatures, i.e., ASPM, AURKA, BUB1, BUB1B, CCNA2, CCNB2, KIF2C, MELK, NCAPG, NCAPH, NUF2, PBK, and TOP2A. These 13 hub genes could behave as potential biomarkers as their overexpression resulted in abnormal cell division leading to tumorigenesis and metastasis in HCC, and this cancer metastasized in the brain, causing GBM. This overexpression resulted in the poor survival of patients in both GBM and HCC. Proper design of suitable inhibitors for these overexpressed hub genes will help in reducing the tumorigenesis and metastasis of HCC, thereby increasing the overall survival outcomes of the patients.

## Figures and Tables

**Table 1:** Table showing the survival analysis results of hub genes in GBM.

Gene	Cox Coefficient	Hazard Ratio (CI)
*ASPM*	0.030	1.58 (0.89–2.79)
*AURKA*	0.041	1.57 (0.93–2.68)
*BUB1*	0.044	1.45 (0.96–2.21)
*BUB1B*	−0.030	1.52 (1.05–2.21)
*CCNA2*	0.011	0.69 (0.48–1.00)
*CCNB2*	0.007	0.73 (0.47–1.15)
*KIF2C*	0.046	0.71 (0.49–1.02)
*MELK*	0.009	1.23 (0.85–1.79)
*NCAPG*	0.010	0.68 (0.47–0.99)
*NCAPH*	0.013	0.73 (0.50–1.05)
*NUF2*	−0.032	1.32 (0.91–1.93)
*PBK*	0.108	1.64 (0.94–2.85)
*TOP2A*	0.035	0.70 (0.49–1.01)

**Table 2:** Table showing the survival analysis results of hub genes in HCC.

Gene	Cox Coefficient	Hazard Ratio (CI)
*ASPM*	0.078	1.50 (1.05–2.13)
*AURKA*	0.020	1.28 (0.90–1.81)
*BUB1*	0.250	1.68 (1.18–2.39)
*BUB1B*	0.268	1.51 (1.06–2.14)
*CCNA2*	0.173	1.56 (1.10–2.21)
*CCNB2*	0.139	1.27 (0.90–1.80)
*KIF2C*	0.278	1.57 (1.10–2.23)
*MELK*	0.278	1.62 (1.14–2.30)
*NCAPG*	0.153	1.31 (0.92–1.85)
*NCAPH*	0.235	1.53 (1.08–2.18)
*NUF2*	0.268	1.48 (1.04–2.11)
*PBK*	0.233	1.47 (1.03–2.08)
*TOP2A*	0.062	1.44 (1.02–2.05)

## Data Availability

The datasets for both GBM and HCC publicly available in Gene Expression Omnibus (GEO). For GBM (normal samples-PRJNA494560 Tumor-samples-PRJNA347513, and for HCC (normal samples-PRJNA494560 and tumor samples-PRJNA414787.

## Supplementary Materials

The following supporting information can be downloaded at https://www.mdpi.com/article/10.3390/diagnostics13050965/s1, Figure S1: MA plot representing the log fold-change against mean expression using DESeq2 dataset (a) MA plot of GBM (b) MA plot of HCC; Figure S2: (a) Figure depicting the normal and cancerous brain tissue taken from Human Protein Atlas (HPA) (b) Figure depicting the normal and cancerous liver tissue taken from Human protein Atlas (HPA); Figure S3: Protein-protein interaction networks of 757 common hub genes between GBM and HCC obtained from DESeq2 analysis. The confidence interval >0.7. Nodes represent proteins and edges represent interaction between proteins; Figure S4 (**a**): Promoter methylation plots of 13 hub genes in GBM Figure S4 (**b**): Promoter methylation of HCC; Figure S5: Differential expression pattern of hub genes of both GBM and HCC combined to demonstrate the expression of diseased tumor samples against the normal samples in both the cases. The expression level shows higher expression of samples in case of GBM as compared to those of GBM; Figure S6: (a) Visualization of genetic alterations of hub genes in GBM using OncoPrint (b) Genetic alterations and their respective alteration frequencies of 13 hub genes in GBM. Green color represents mutations, red color represents amplification, and blue color represents deep deletion. All the hub genes have either missense mutation or nonsense mutation; Figure S7: Copy number variations of 13 hub genes in GBM; Figure S8: (a) Visualization of genetic alterations of hub genes in HCC using OncoPrint (b) Genetic alterations and their respective alteration frequencies of 13 hub genes in HCC. Green color represents mutations, red color represents amplification, and blue color represents deep deletion. All the hub genes except CCNB2 and KIF2C have either missense mutation or nonsense mutation; Figure S9: Copy number alterations of hub genes in HCC Table S1: Table showing the detailed analysis of genetic alterations in GBM Table S2: Table showing the detailed analysis of genetic alterations in HCC.
