# Supplementary material for: Identification of Prognostic Biomarkers for Suppressing Tumorigenesis and Metastasis of Hepatocellular Carcinoma through Transcriptome Analysis
Source: Diagnostics (Basel). 2023 Mar 3;13(5):965. doi: 10.3390/diagnostics13050965 (PMC10001411; doi:10.3390/diagnostics13050965)

Supplementary Figure S1 MA plot representing the log fold-change against mean expression using DESeq2 dataset (a) MA plot of GBM (b) MA plot of HCC

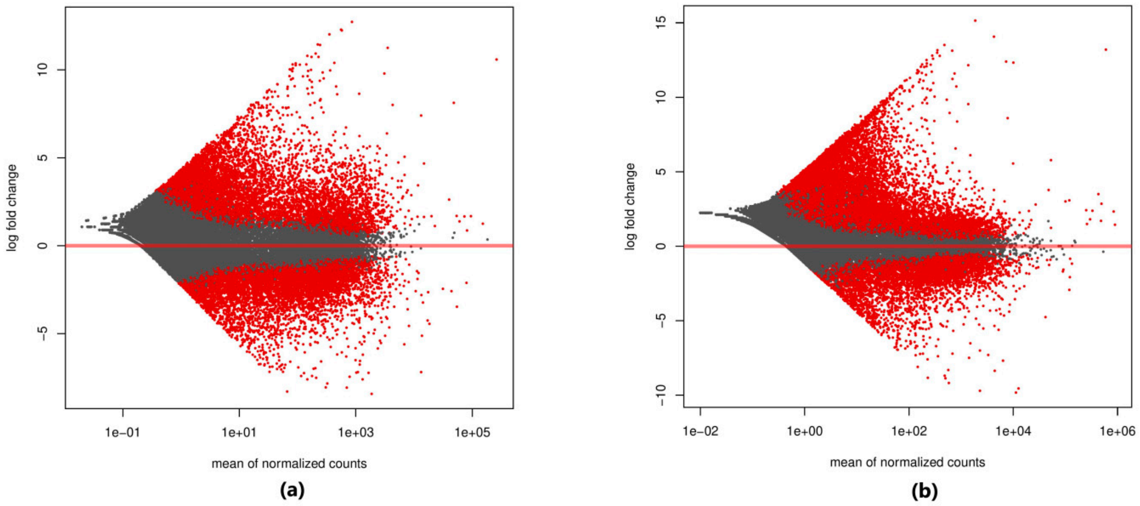

Supplementary Figure S2 (a) Figure depicting the normal and cancerous brain tissue taken from Human Protein Atlas (HPA) (b) Figure depicting the normal and cancerous liver tissue taken from Human protein Atlas (HPA)

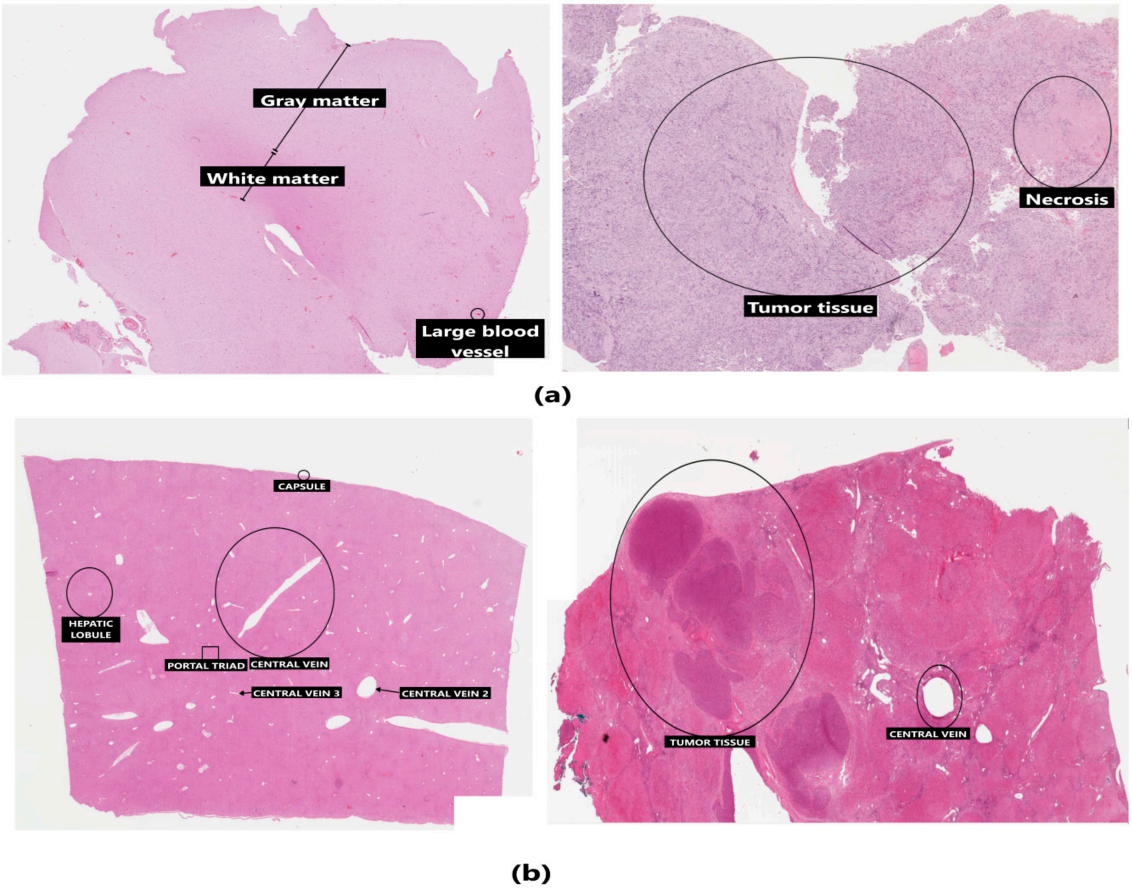

Supplementary Figure S3 Protein-protein interaction networks of 757 common hub genes between GBM and HCC obtained from DESeq2 analysis. The confidence interval > 0.7. Nodes represent proteins and edges represent interaction between proteins.

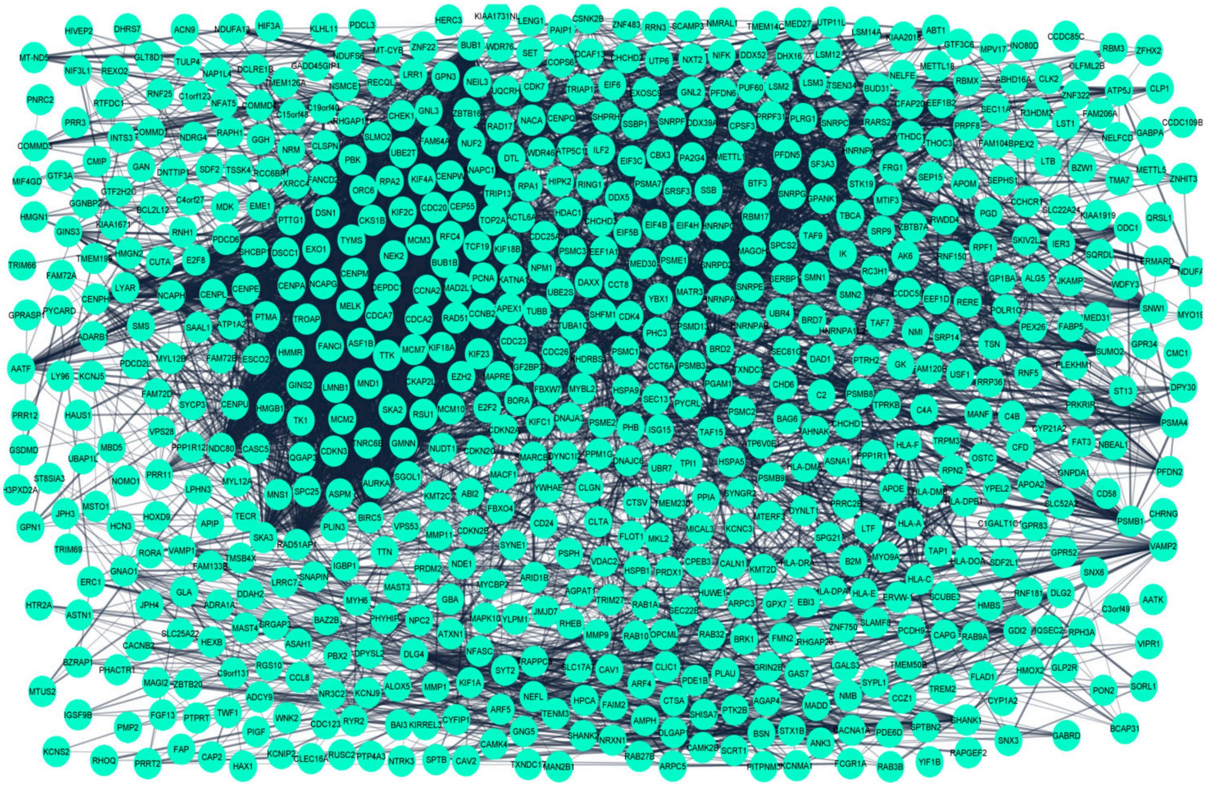

Supplementary Figure S4 (a) Promoter methylation plots of 13 hub genes in GBM

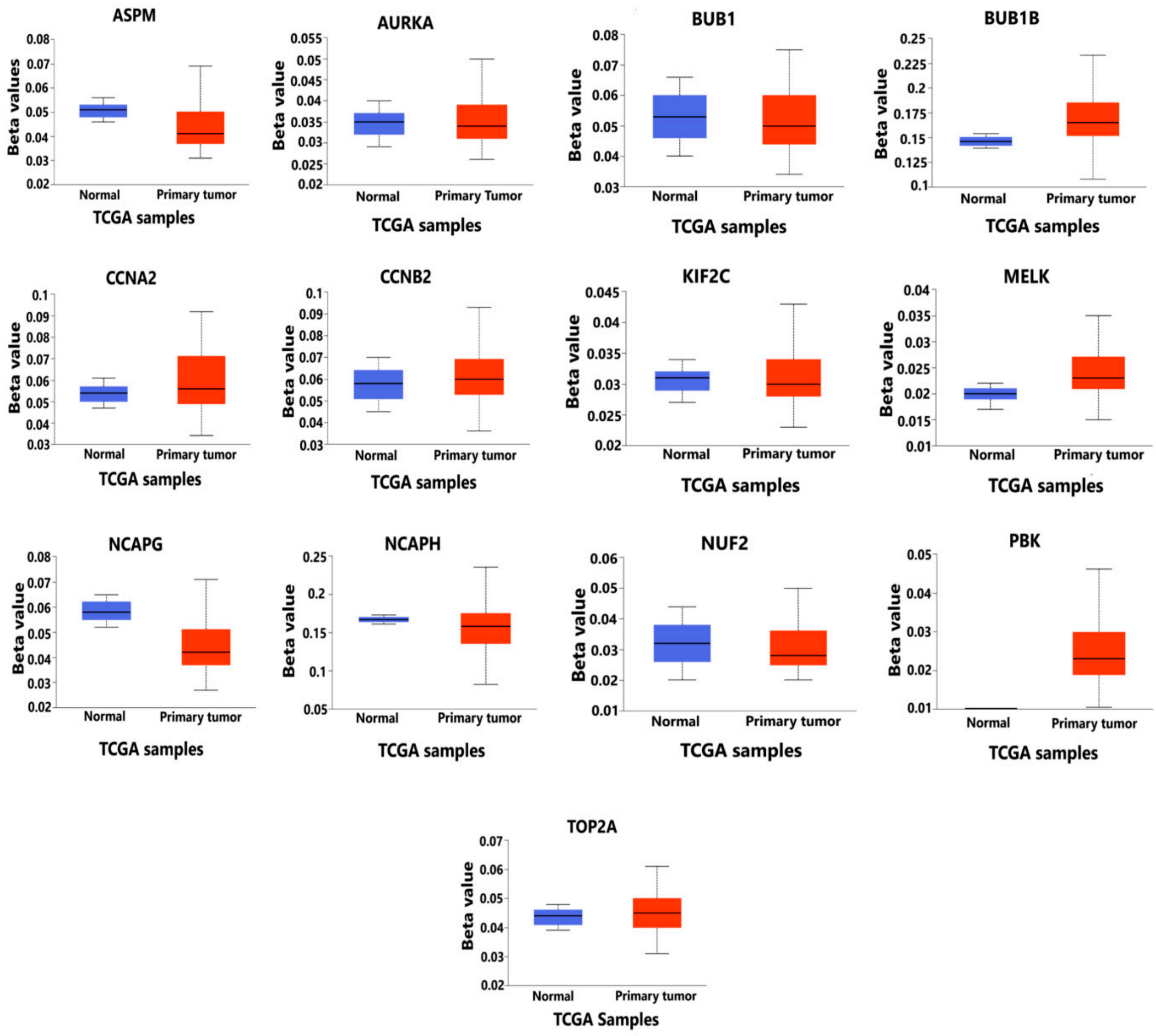

Supplementary Figure S4 (b) Promoter methylation of HCC

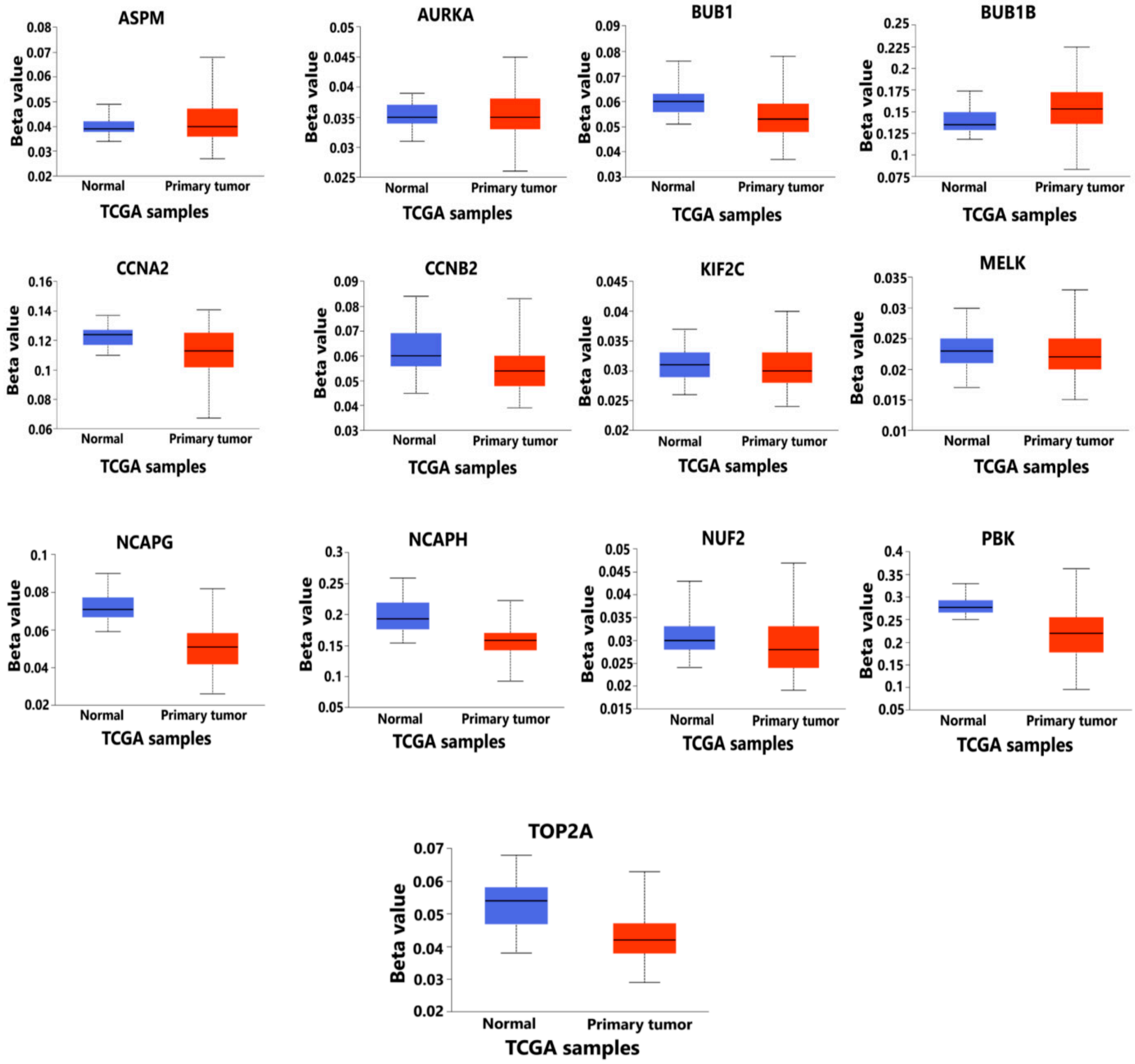

Supplementary Figure S5 Differential expression pattern of hub genes of both GBM and HCC combined to demonstrate the expression of diseased tumor samples against the normal samples in both the cases. The expression level shows higher expression of samples in case of GBM as compared to those of GBM

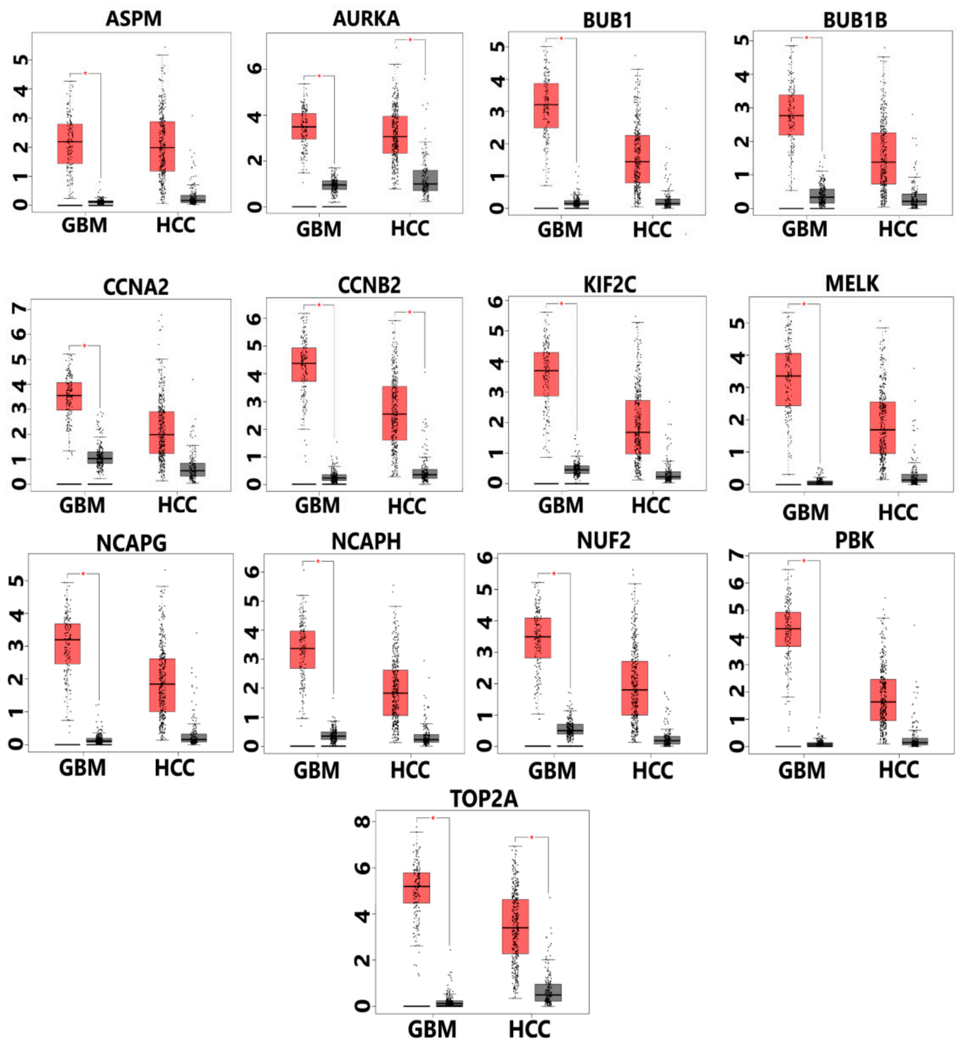

Supplementary Figure S6 (a) Visualization of genetic alterations of hub genes in GBM using OncoPrint (b) Genetic alterations and their respective alteration frequencies of 13 hub genes in GBM. Green color represents mutations, red color represents amplification, and blue color represents deep deletion. All the hub genes have either missense mutation or nonsense mutation

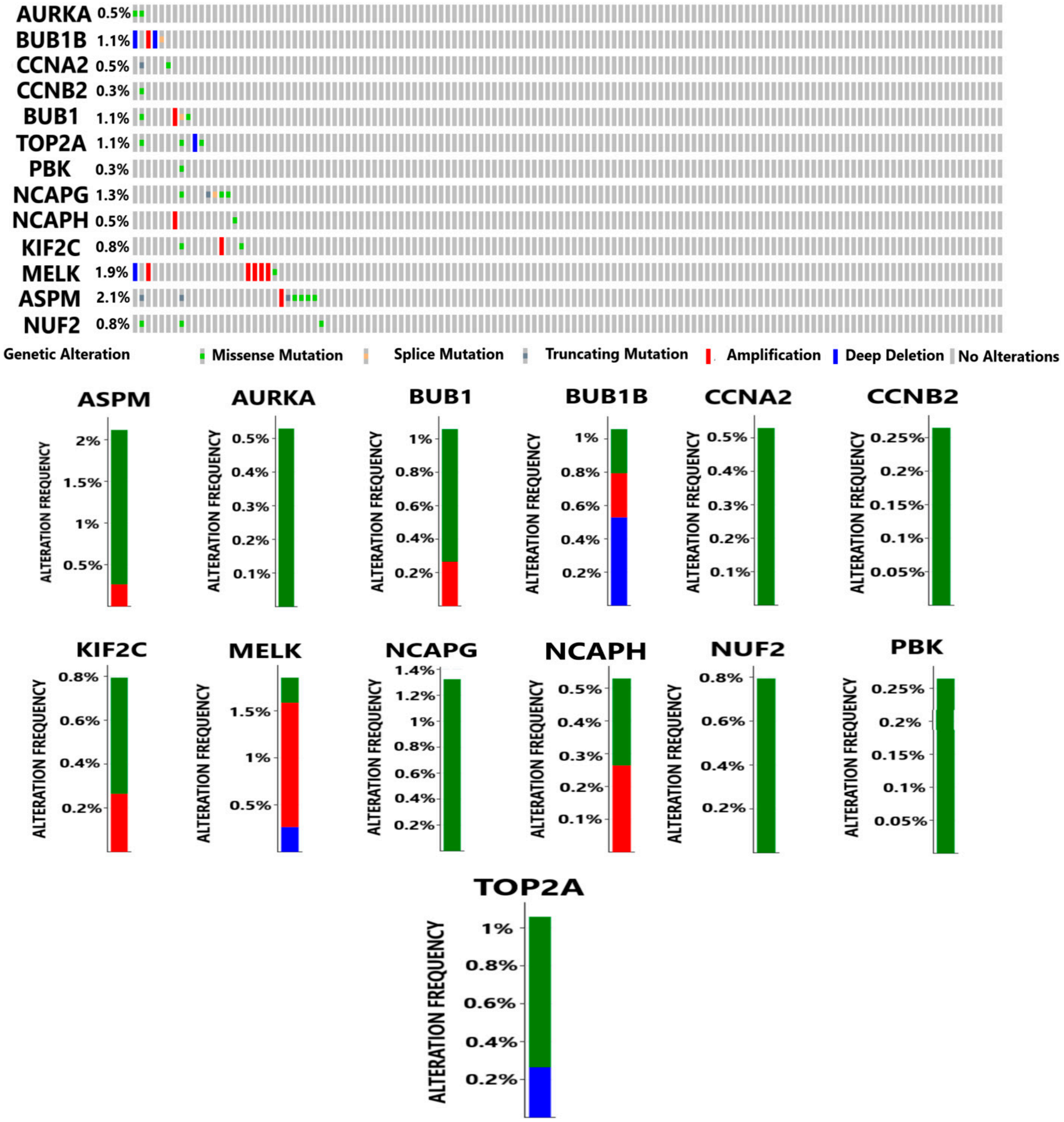

Supplementary Figure S7 Copy number variations of 13 hub genes in GBM

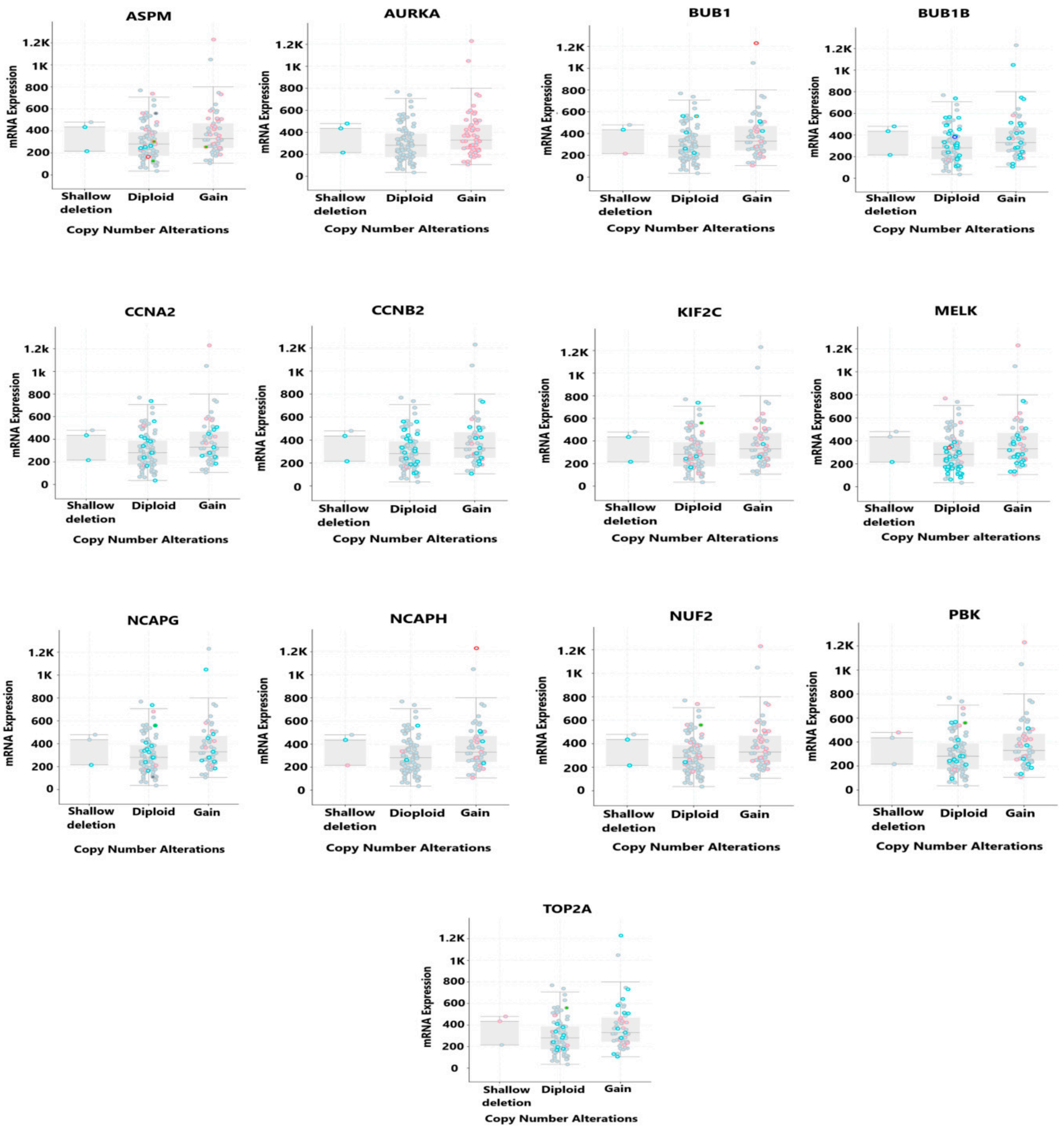

Supplementary Figure S8 (a) Visualization of genetic alterations of hub genes in HCC using OncoPrint (b) Genetic alterations and their respective alteration frequencies of 13 hub genes in HCC. Green color represents mutations, red color represents amplification, and blue color represents deep deletion. All the hub genes except CCNB2 and KIF2C have either missense mutation or nonsense mutation.

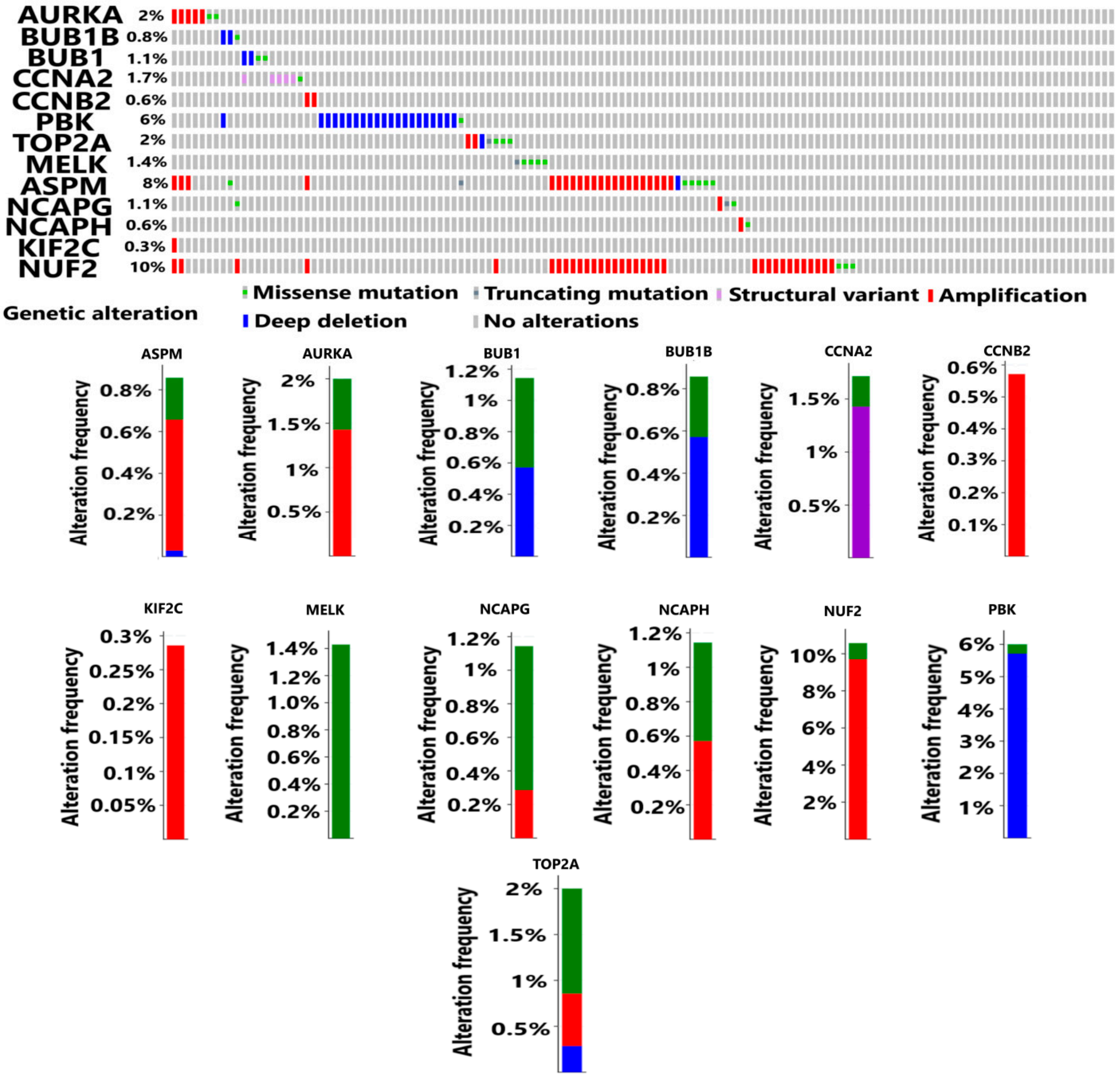

Supplementary Figure S9 Copy number alterations of hub genes in HCC

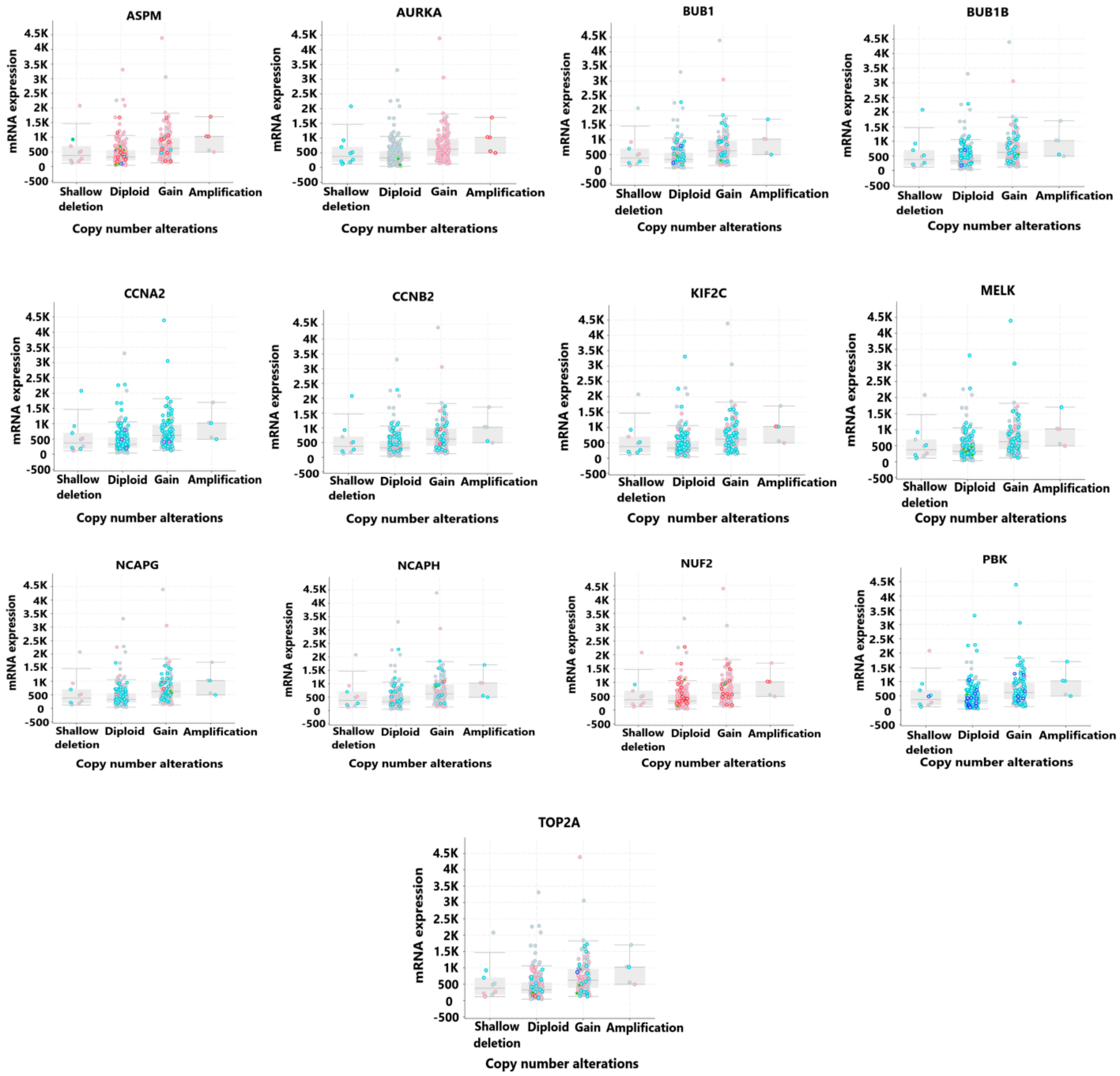

Supplement: Supplementary file 1 [file diagnostics-13-00965-s001.zip › diagnostics-2132760-Supplementary figures.pdf]
