# Supplementary material for: Identification of Prognostic Biomarkers for Suppressing Tumorigenesis and Metastasis of Hepatocellular Carcinoma through Transcriptome Analysis
Source: Diagnostics (Basel). 2023 Mar 3;13(5):965. doi: 10.3390/diagnostics13050965 (PMC10001411; doi:10.3390/diagnostics13050965)
Supplement: Supplementary file 1 [file diagnostics-13-00965-s001.zip › diagnostics-2132760-Supplementary tables.pdf]

Supplementary Table S1 Table showing the detailed analysis of genetic alterations in GBM

S. No.	*Gene Name*	*Types of Genetic Alterations (%)*	*Post Translational Modifications (PTMs)*	*Mutation Type*	*Mutation Site*	*Copy Number Alteration*
1	ASPM	Mutation (2%)<br>Amplification (6.29%)<br>Deep Deletion (0.29%)	Phosphorylation	Splice	L642=	Diploid
			Phosphorylation	Nonsense	R3152*	Diploid
			Phosphorylation	Nonsense	R3244*	Diploid
			Phosphorylation, Acetylation, Ubiquitination Methylation	Nonsense	E2012*	Diploid
			NA	Missense	R854H	Diploid
			NA	Missense	E918K	Diploid
			Phosphorylation, Acetylation, Ubiquitination	Missense	S270P	Gain
			NA	Missense	A3137D	Gain
			Phosphorylation, Acetylation, Ubiquitination Methylation	Missense	T2339P	Diploid
			Phosphorylation, Acetylation, Ubiquitination Methylation	Missense	R2442Q	Diploid
			Phosphorylation	Missense	A3013V	Diploid
			Phosphorylation, Acetylation, Ubiquitination Methylation	Missense	K2586Q	Diploid
					A2152T	Diploid
			Phosphorylation, Acetylation, Ubiquitination Methylation	Missense		
			Phosphorylation, Acetylation, Ubiquitination Methylation	Missense	D876N	Diploid
			NA	Missense	L817F	Diploid
			NA	Missense	K2081I	Diploid
			NA	Missense	K884M	Diploid
2	AURKA	Mutation (0.57%)<br>Amplification	Phosphorylation	Missense	G11R	Diploid
			Phosphorylation, Acetylation	Missense	R56H	Diploid

		(1.43%)	Phosphorylation	Missense	E336D	Diploid
3	BUB1	Mutation (0.57%)<br>Deep Deletion (0.57%)	Phosphorylation<br>NA<br>Phosphorylation, Ubiquitination<br>NA<br>Phosphorylation	Missense<br>Splice<br>Missense<br>Missense<br>Missense	C698Y<br>X1021_splice<br>G533E<br>F818C<br>T452M	Diploid<br>Shallow Deletion<br>Shallow Deletion<br>Shallow Deletion<br>Shallow Deletion
4	BUB1B	Mutation (0.29%)<br>Deep Deletion (0.57%)	NA	Splice	X950_splice	Diploid
5	CCNA2	Mutation (0.29%)<br>Structural variant (1.43%)	Phosphorylation, Acetylation, Ubiquitination<br>NA	Missense<br>Nonsense	A25V<br>E269*	Gain<br>Gain
6	CCNB2	Amplification (0.57%)	Phosphorylation	Missense	P80S	Shallow Deletion
7	KIF2C	Amplification (0.29%)	Phosphorylation<br>Acetylation<br>Phosphorylation, Ubiquitination, Methylation	Missense<br>Missense<br>Missense	A648T<br>E684K<br>Q508H	Diploid<br>Diploid<br>Gain
8	MELK	Mutation (1.43%)	Phosphorylation	Missense	A315S	Diploid
9	NCAPG	Mutation (0.86%)<br>Amplification (0.29%)	Phosphorylation, Ubiquitination<br>Phosphorylation<br>Phosphorylation<br>NA<br>Phosphorylation	Missense<br>Nonsense<br>Splice<br>Missense<br>Missense	A113S<br>A364L<br>X373_splice<br>L444V<br>S467*	Gain<br>Diploid<br>Diploid<br>Shallow Deletion<br>Diploid
10	NCAPH	Mutation (0.29%)<br>Amplification (0.29%)	NA	Missense	Q704K	Diploid

11	NUF2	Mutation (0.79%)<br>Amplification (	Phosphorylation, Ubiquitination<br>Ubiquitination<br>Phosphorylation	Missense<br>Missense<br>Missense	S247Y<br>Q290H<br>D435N	Diploid<br>Diploid<br>Diploid
12	PBK	Mutation (0.26%)	Phosphorylation	Missense	Q291K	Diploid
13	TOP2A	Mutation (0.79%)<br>Deep Deletion (0.26%)	Phosphorylation, Acetylation, Sumoylation<br>Phosphorylation, Acetylation, Sumoylation<br>Acetylation, Ubiquitination, Sumoylation<br>Phosphorylation, Acetylation, Methylation, Sumoylation<br>Phosphorylation, Acetylation, Sumoylation	Missense<br>Missense<br>Missense<br>Missense<br>Missense	T38A<br>S53Y<br>V1076F<br>S1483L<br>K1492N	Diploid<br>Diploid<br>Diploid<br>Diploid<br>Diploid

Supplementary Table S2 Table showing the detailed analysis of genetic alterations in HCC

S. No.	*Gene Name*	*Types of Genetic Alterations (%)*	*Post Translational Modifications (PTMs)*	*Mutation Type*	*Mutation Site*	*Copy Number Alteration*
1	ASPM	Mutation (2%)<br>Amplification (6.29%)<br>Deep Deletion (0.29%)	Phosphorylation<br>Phosphorylation<br>Phosphorylation<br>NA<br>NA<br>Acetylation<br>NA<br>NA	Missense<br>Missense<br>Missense<br>Missense<br>Missense<br>Missense<br>Missense<br>FS ins	T111A<br>K610E<br>F645L<br>R792W<br>I1051T<br>Y2007S<br>G2156R<br>Q2620Tfs*17	Diploid<br>Gain<br>Diploid<br>Diploid<br>Gain<br>Gain<br>Shallow Deletion<br>Gain
2	AURKA	Mutation (0.57%)<br>Amplification (1.43%)	Phosphorylation<br>Phosphorylation	Missense<br>Missense	S83N<br>R180K	Diploid<br>Diploid
3	BUB1	Mutation (0.57%)<br>Deep Deletion	NA<br>NA	Missense<br>Missense	T805I<br>S950R	Diploid<br>Diploid

		(0.57%)				
4	BUB1B	Mutation (0.29%)<br>Deep Deletion<br>(0.57%)	NA	Missense	Q460L	Diploid
5	CCNA2	Mutation (0.29%)<br>Structural variant<br>(1.43%)	NA	Missense	N129K	Diploid
6	CCNB2	Amplification<br>(0.57%)	NA	No mutation		
7	KIF2C	Amplification<br>(0.29%)	NA	No mutation		
8	MELK	Mutation (1.43%)	NA<br>Phosphorylation<br>NA<br>NA	Missense<br>Missense<br>Missense<br>Missense	R53L<br>I237S/V<br>V287I<br>Y638Sfs*4	Diploid<br>Diploid<br>Diploid<br>Diploid
9	NCAPG	Mutation (0.86%)<br>Amplification<br>(0.29%)	NA<br>NA<br>Phosphorylation,<br>Ubiquitination	Missense<br>Nonsense<br>Missense	L96F<br>S574*<br>Y850H	Diploid<br>Diploid<br>Diploid
10	NCAPH	Mutation (0.29%)<br>Amplification<br>(0.29%)	NA	Missense	T264A	Diploid
11	NUF2	Mutation (0.86%)<br>Amplification<br>(9.71%)	NA<br>NA<br>NA	Missense<br>Missense<br>Missense	E13D<br>G385D<br>Y445C	Gain<br>Gain<br>Gain
12	PBK	Mutation (0.29%)<br>Deep Deletion<br>(5.71%)	NA	Missense	E303V	Diploid
13	TOP2A	Mutation (1.14%)<br>Amplification<br>(0.57%)<br><br>Deep Deletion<br>(0.29%)	Phosphorylation<br>NA<br>NA<br><br>NA	Nonsense<br>Missense<br>Missense<br><br>Missense	R450*<br>T689N<br>R877W<br><br>T1315K	Diploid<br>Gain<br>Diploid<br><br>Diploid
